# Predicting Public Attitudes Toward Gene Editing of Germlines: The Impact of Moral and Hereditary Concern in Human and Animal Applications

**DOI:** 10.3389/fgene.2018.00704

**Published:** 2019-01-09

**Authors:** Christine Critchley, Dianne Nicol, Gordana Bruce, Jarrod Walshe, Tamara Treleaven, Bernard Tuch

**Affiliations:** ^1^Centre for Law and Genetics, School of Law, University of Tasmania, Hobart, TAS, Australia; ^2^Department of Statistics, Data Science and Epidemiology, Swinburne University of Technology, Melbourne, VIC, Australia; ^3^Discipline of Physiology, Faculty of Medicine and Health, School of Medical Sciences, The University of Sydney, Sydney, NSW, Australia

**Keywords:** gene editing, public opinion survey, cell type, morality, germline, CRISPR-Cas9, multilevel modeling, religiosity

## Abstract

**Background and Objective:** New and more efficient methods of gene editing have intensified the ethical and legal issues associated with editing germlines. Yet no research has separated the impact of hereditary concern on public attitudes from moral concern. This research compares the impact these two concerns have on public attitudes across five applications including, the prevention of human disease, human and animal research, animals for the use of human food and the enhancement of human appearance.

**Methods:** A sample of 1004 Australians responded to either a telephone (*n* = 501; randomly selected) or online survey (*n* = 503; sourced by Qualtrics). Both samples were representative in terms of States and Territories as well as gender (51% female), though the online sample was younger (*M* = 40.64, *SD* = 16.98; Range = 18–87) than the telephone sample (*M* = 54.79, *SD* = 18.13; Range = 18–96). A 5 (application) by 3 (type of cell) within groups design was utilized, where all respondents reported their level of approval with scientists editing genes across the 15 different contexts. Multilevel modeling was used to examine the impact of moral (embryo vs. germ) and hereditary (germ vs. somatic) concern on attitudes across all applications.

**Results:** Australians were comfortable with editing human and animal embryos, but only for research purposes and to enhance human health. The effect of moral concern was stronger than hereditary concern, existing in all applications except for the use of animals for human purposes. Hereditary concern was only found to influence attitudes in two applications: improving human health and human research. Moral concern was found to be accentuated amongst, women, more religious individuals and those identifying as Australian, while hereditary concern was strongest amongst non-Australians, those with stronger trust in scientists, and more religious respondents.

**Conclusion:** Moral and hereditary concerns are distinct, and require different approaches to public education, engagement and possibly regulation. Further research needs to explore hereditary concern in relation to non-human applications, and the reasons underlying cultural and gender differences.

## Introduction

Since its first emergence in 2012, CRISPR-Cas9 (clustered regularly interspaced short palindromic repeats) has become the most dominant of a suite of new generation of genetic editing methods. Extensive media and scholarly comment have declared that CRSISPR-Cas9 will “supercharge” our understanding of biological life. A multitude of applications and “breakthroughs” are predicted, including the elimination of pests and diseases, advances in food production, improved human health, and even changes to the course of evolution (Funnell, 2016; Park, 2016; Doudna and Sternberg, 2017). These claims are not new, with the promise of cures for diseases and more nutritious food crops, for example, existing since the advent of genetic engineering in the 1970's (Hogan, 2016). Relative to earlier methods however, the reduced cost, enhanced efficiency and ease of use mean that potentially any molecular biology laboratory can use CRISPR-Cas9 to insert, replace or delete sections of DNA in any living organism.

There are indeed signs that an explosion in CRISPR-Cas9 research has already commenced, with Science magazine's 2015 “Breakthrough of the Year” already being employed to alter crops (Cai et al., 2015) and animals (Whitworth et al., 2014) for food production and the first clinical trials on humans beginning in 2016 (Cyranoski, 2016). The acceleration of CRISPR-Cas9 and other gene editing techniques have however outpaced legal and regulatory guidelines, especially in relation to heritable changes. Scientists are therefore concerned that outdated legislation and guidelines established prior to 2012 will impede progress, particularly the development of much needed human genetic therapies (Nicol et al., 2017). For example, modifications to the genome within human embryos and germ cells that can be theoretically inherited are considered especially promising in eradicating genetic disorders and understanding human development, yet this practice is currently restricted (in varying degrees) in many jurisdictions (Isasi and Knoppers, 2015; Isasi et al., 2016; Nicol et al., 2017).

In most jurisdictions, legislation attempting to guide the ethical use of genetic engineering and human embryonic research prior to 2012 was driven by political, economic, religious and community pressure to protect the moral status of the human embryo, and avoid potential dystopian outcomes such as human clones, designer babies, genetic inequity and eugenics (Nuffield Council on Bioethics., 2016). Legislation associated with the modification of animal and plant genes was also associated with pressure to prevent disruption to our ecosystem, environmental damage and, importantly, to ensure the safety of genetically modified organisms for human consumption (Tsatsakis et al., 2017). With the availability of new gene editing methods, these ethical legal and social concerns have intensified, especially in relation to heritable edits of the human genome (Nuffield Council on Bioethics., 2016). In 2014 the scientific community was forced to confront these issues when Liang et al. (2015) modified the gene responsible for a potentially fatal blood disorder in 86 non-viable human embryos. The work was rejected by both the prestigious journals *Nature* and *Science* partly due to ethical issues, leading to a media frenzy and calls amongst the scientific community for a moratorium on editing human germlines (Cyranoski and Reardon, 2015). Acknowledging the ethical concerns as well as the potential benefits, the US National Academies of Sciences, Engineering, and Medicine convened the Committee on Human Gene Editing: Scientific, Medical, and Ethical Considerations to review the science, ethics and governance of human genome editing. A comprehensive report followed, culminating in guiding principles for governance, and recommendations for the oversight and use of human genome editing. While acknowledging that “basic laboratory research involving genome editing of human cells and tissues is critical to advancing biomedical science” (National Academies of Sciences, 2017, p4), the report recommended that both somatic and germline editing should only occur for the purpose of preventing disease or disability. Germline editing was considered only acceptable if there are compelling reasons and “a stringent oversight system able to limit uses to specified criteria” (p13).

The NASEM report also recommended an ongoing “reassessment of both health and societal benefits and risks, with broad ongoing participation and input by the public” (p178). Other scholars agree arguing, that “public sentiment has considerable influence over allocation of resources, political policy and participation rates in studies, all of which affect the course of research” (McCaughey et al., 2016, p. 571). More specific to gene editing, a survey of 301 attendees (74% basic scientists) at a plenary entitled “Scientific, Clinical and Ethical Implications of Genome Editing” at an American Heart Association conference in 2017 found that 72% indicated that they would not support “clinical applications of human germline genome editing if the public was not asked about their opinion on the issue” (Musunuru et al., 2017, p. 2). Lawyers and ethicists also advocate public engagement to prevent a potential backlash against media hype associated with germline editing obscuring or biasing the potential benefits gained from gene editing somatic cells within the clinic (Lanphier et al., 2015; Nicol et al., 2017). The aim of this research was therefore to conduct a national survey to extend our knowledge of attitudes toward gene editing, inform policy and, importantly, provide a voice for the public.

A first step in considering public opinion is to determine the extent of support and its drivers. Apart from a handful of studies, little is currently known about lay perceptions of gene editing of germline cells and embryos in humans or animals. To date, only five peer reviewed surveys have been published post 2012 (McCaughey et al., 2016; Gaskell et al., 2017; Rose et al., 2017; Weisberg et al., 2017; Scheufele et al., 2018), reporting results that coincide with earlier findings that people tend to support genetic and genomic research generally (including genetic engineering/modification) (Lucht, 2015) if it is safe and the purpose is altruistic (e.g., to improve human health) (Henneman et al., 2006; Condit, 2010). The first peer reviewed study within the context of CRISPR also found more support for therapeutic relative to enhancement applications, but support for using embryos depended on the purpose (McCaughey et al., 2016). Amongst a young (Median age = 24) predominately male (62.1%) sample of 12,562 social media users across 185 countries (primarily the US, UK, and China), McCaughey et al. found that the majority supported editing cells in embryos to prevent a debilitating (63%) or life threatening disease (63%) (And where “all future generations would not have the disease”), and within children or adults to cure a debilitating (59%) or life threatening disease (59%) (And where “the disease could still be passed on to their children”). Support was low for editing genes within an embryo to alter any non-disease characteristics such as memory, eye color or height (27%)^1^. They also found significantly greater support for all applications amongst males, younger people and those without a religious affiliation. Support for editing genes to treat diseases was also significantly higher for those who had finished a tertiary education and for those in wealthier countries^2^. Self -reported wealth, however, was not associated with support, nor was a family history of a monogenic Mendelian disease.

Gaskell et al. (2017) also found significantly high support in 2017 for therapeutic, relative to enhancement applications. An online panel sample of 11,716 respondents across the US and UK, Austria, Denmark, Germany, Hungary, Iceland, Italy, the Netherlands, Portugal, and Spain was used to investigate the perceived morality of different applications of gene editing. They also found strong support for therapeutic applications and low support for the purposes of enhancement across all countries. Specifically altering “the gene sequence in some affected cells” to improve symptoms of dementia in an adult patient (Median = 8 from a possible 11 point scale) and in an unborn child genetically at risk for dementia (Median = 6) were viewed as more morally acceptable than enhancing memory and learning capacities in a healthy adult (Median = 2) or unborn child (Median = 0). Altering the genes of adults was also viewed as more morally acceptable than altering the genes of an unborn child, again in all countries. The level of moral acceptability was not significantly associated with age or education, but females were generally less supportive than males of all applications.

A recent (late 2016 and early 2017) national survey of 1600 U.S. adults found similar results (Scheufele et al., 2018). Higher approval was reported for therapeutic applications (e.g., human gene editing to treat human medical conditions or restore health) than for human enhancement (e.g., human gene editing to enhance or improve human abilities). Support was similar and relatively high for both somatic and germline therapies. More religious respondents and women were less supportive of gene editing for both applications.

A fourth study utilized 1,249 online panel members (recruited by Amazon's Mechanical Turk) to investigate the impact of metaphors or how genetic editing is framed (i.e., genetic editing, genetic engineering, genetic surgery, and genetic hacking) on beliefs in whether we should “be actively researching these technologies” (−3 = Absolutely not, 0 = Unsure, 3 = Absolutely yes) (Weisberg et al., 2017). They also explored whether exposure to the risks of gene editing^3^ influenced beliefs in whether the technology should be used. They found, amongst the relatively young (Median = 37.2 years), left wing and educated sample of predominantly males, strong general support for gene editing (*M* = 1.65, *SD* = 1.32). There were no differences in relation to framing, but the study found support decreased for respondents who received the vignette containing the risks (*M* = 1.43, *SD* = 1.36) relative to those who did not (*M* = 1.87, *SD* = 1.24). Men, younger people, non-African Americans and those with more than 4 years of college education and reporting a more left wing political ideology were significantly more supportive. Having a genetic disease or being related to someone with a genetic disease was not associated with support.

Finally, two studies reporting on participants of public scientific engagement events also reported strong support. The first was conducted with 183 members of the US public who participated in the 2015 Wisconsin Science Festival. A total of 34 participants attended a panel discussion including scientists, academics and ethicists on human gene editing titled “Designer Genes: Should We Be Able to Edit Our Genomes?” A survey administered to the participants before (*n* = 34) and after (*n* = 26) the festival found that participants' generally believed that “genetic editing is morally acceptable” (*M* = 3.38, *SD* = 1.00; 1 = Strongly Disagree−5 = Strongly Agree), and that this belief increased significantly after the panel discussion (*M* = 4.00, *SD* = 0.71). Knowledge of gene editing also significantly increased after the event as well as perceptions that there are both benefits and risks to society.

Another evaluation of 57 public attendees to an Australian Q and A expert panel discussion on gene editing (as part of the Sydney Science Festival) also found support for editing the genes of adults and children for clinical trials, human embryos for research, and animals for research (Treleaven and Tuch, 2018). The majority also approved of the use of gene editing within human embryos to improve health and where the embryo is allowed to develop into a baby, although support was lower for this than other applications. Respondents did not agree that altering human embryos for enhancement purposes (i.e., gender selection, eye color) was acceptable. Although support appeared to increase for altering the genes of embryos after the discussion, the differences were not significant.

Research examining public opinion relating to new forms of genomic editing is therefore in its infancy. Emerging evidence appears to suggest support varies according to the application and particularly in relation to editing within human embryos, but this also appears to vary alongside individual differences unique to particular samples (i.e., those derived from online panels or social media). There has also been no direct investigation into the relative influence of both the source of cell (human, animal, embryo, reproductive, somatic) and the purpose of editing (to prevent disease, research purposes, enhance attributes). In particular, there has been no attempt to separate the effects of editing germ cells from those that occur within embryos. Establishing greater public awareness of the distinction between germline and somatic cell editing has been repeatedly emphasized (Lanphier et al., 2015; Cartier-Lacave et al., 2016; Olson, 2016), yet we know little about whether public support is influenced by concerns about inherited edits being passed on to offspring. Previous studies have only compared support for gene editing somatic to embryonic cells (McCaughey et al., 2016; Gaskell et al., 2017) which may confound concern with inherited edits with moral or religious values attached to human embryos (Allum et al., 2017).

We therefore directly investigated for the first time whether public support for gene editing differs across somatic, germ cells and embryos suggesting distinct underlying concerns. Differences between germ and embryonic cells were defined as concern for the moral status of the human embryo (referred throughout as moral concern), while variation in support for somatic compared to germ cells was considered concern for passing edits on (referred to throughout as hereditary concern). Using both a probabilistic population survey (randomly digit dialed telephone interview) as well as an identical online panel survey (sourced by Qualtrics), we also examined the effect of cell type on attitudes in combination with different applications: for research purposes only; to improve health or prevent disease; in animals for human use; and for human enhancement (e.g., to change appearance). Finally we also explored the role of demographic and individual difference variables (i.e., trust in scientists, political orientation, knowledge) in explaining moral or hereditary concerns and general support for gene editing. Whilst current research suggests support will be lower for enhancement than for improving human health, we had no prior expectations of which factors and what combinations would be most important in predicting support. Nevertheless, the ability to directly compare multiple factors within the same study was expected to shed light on which issues cause most concern, and thereby provide valuable direction for policy makers keen to address public confidence and trust in regulatory procedures governing gene editing, and on where to direct educational strategies.

## Materials and Methods

### Participants and Procedure

A total of 1004 respondents were surveyed with an identical measurement instrument, 501 via a computer assisted telephone interview (CATI) and 503 via an online panel sample (OLP) sourced through Qualtrics^4^ Both samples were stratified by state and territory, with the OLP being also stratified by gender. The CATI survey consisted of randomly generated mobile (43.5%) and landline (56.5%) numbers sourced by Sampleworx^5^ The response rates for the CATI survey according to the American Association for Public Opinion Research (2009) definitions and calculations (i.e., RR1–RR4) ranged from 6.0 to 9.0% with a cooperation rate of between 13.8 and 15.4%.

CATI and OLP respondents were not significantly (at *p* < 0.05) different in terms of state and territory, gender (CATI = 51.3% female; OLP = 51.1%), and political orientation (CATI: *M* = 5.00, *SD* = 2.17; OLP: *M* = 4.97, *SD* = 2.12; Range: 1 = Left wing−10 = Right wing). The CATI sample (*M* = 54.79, *SD* = 18.13; Range = 18–96) was however significantly (*p* < 0.001) older than the OLP sample (*M* = 40.64, *SD* = 16.98; Range = 18–87), more likely to have a university degree (CATI: 47.9%; OLP: 36.0%), and less likely to state that their ethnicity was Australian (CATI = 76%; OLP = 83.3%). The OLP sample was more likely to be working full (38.2%: CATI = 28.9%) and part time (20.9%; CATI = 15.7%), engaged in home duties (9.9%; CATI = 3.4%) and unemployed (10.9%; CATI = 6.8%), while the CATI sample were more likely to be retired (36.5%; OLP = 12.5%). The CATI sample also reported being significantly (*p* < 0.001) more spiritual (CATI: *M* = 2.67, *SD* = 1.17; OLP: *M* = 2.33, *SD* = 1.06; Range = 1 = Spirituality is not at all important−4 = Very important), though there were no differences in church attendance (apart from weddings, baptisms or funerals) (*p* = 0.877) or trust in the churches (*p* = 0.10). There were no significant differences between samples in terms of their self-reported knowledge of gene editing or trust in scientists. Both groups demonstrated relatively low knowledge on a 10-point scale where 0 = I know nothing about gene editing and 10 = I know a great deal about gene editing (CATI: *M* = 2.80, *SD* = 2.57; OLP: *M* = 2.91, *SD* = 2.71). Trust is scientists was relatively high for both groups (CATI: *M* = 3.83, *SD* = 0.99; OLP: *M* = 3.70, *SD* = 1.05) in response to being asked “how much do you trust scientists” (0 = Don't trust at all−5 = Trust a very great deal) (see Supplementary Materials for actual wording of all questions).

### Materials

The survey questions were included in the 2017 Swinburne National Technology and Society Monitor, and were identical for the CATI and online sample. Before introducing the topic of gene editing, all respondents were asked about their knowledge of gene editing and were then read the following information:

“Genome editing is when scientists deliberately alter the genes in a living cell to change how a gene functions. So far most uses of gene editing have been for research purposes only, however there are many possible applications. For example, it could be used to alter the genes of plants and animals to increase resistance to disease. It could also be used to develop new drugs, prevent the inheritance of diseases, and determine the attributes of babies.”

Questions were designed to vary across the type of application (improving health, research only, changing appearance and animals for food) and cell source (embryo, germ cell, somatic cell). Respondents were first asked about cells or embryos where the edit could be inherited. Specifically they were asked, “The first lot of scenarios relate to editing genes that could potentially lead to the change being inherited (passed onto another human or animal).” They were then presented with 10 different options (described below) that varied by source and aim, and asked, “For each of the following do you agree with editing the genes of:” The presentation of each option was random across respondents, and the response options were: Strongly Disagree = 1, Disagree = 2, Agree = 3, Strongly Agree = 4, and Unsure.

In this section, two items assessed the application of improving health or preventing disease: “Human embryos [reproductive cells (i.e., egg or sperm)] to improve health or prevent disease.” For research purposes four items were used to assess attitudes across embryos and reproductive cells and animal and humans. That is, “Human [Animal] embryos for research purposes only” and “Human [Animal] reproductive cells (egg or sperm) for research purposes only.” Enhancing attributes was assessed by two items: “Human embryos [reproductive cells (i.e., egg or sperm)] for reasons of producing a baby with certain genes e.g., for hair color, gender selection.” Finally, two items measured differences in source across the aim animals for human purposes, “Animal embryos [reproductive cells (i.e., egg or sperm)] for human purposes (e.g., improving the quality of beef).

Participants were then asked about somatic cells. Specifically, interviewers read out the following:

“The next lot of scenarios relate to editing the genes within a somatic/body cell that cannot pass on the change to another human or animal. For each of the following again, let us know the extent to which you agree or disagree with editing the genes of:”

Six scenarios were randomly presented that assessed attitudes across the five aims. That is, “A human body cell (e.g., eye or heart cell) to improve health or prevent disease (e.g., blindness)?”, “A human [animal] body cell for research purposes only,” “A human body cell to change one's appearance,” and “An animal body cell to alter its characteristics for human purposes (e.g., leaner beef in cows).”

## Results

### Preliminary Analyses

To check for significant differences across the CATI and OLP samples, a series of one way analyses of covariance (ANCOVA) were computed for all 15 scenarios using SPSS Version 25. The demographic differences between the two samples determined the covariates since differences in support may be due to differences in age, education or ethnicity for example rather than because of the different survey administration. A series of binary logistic regression equations suggested that the differences in employment status across CATI and OLP respondents was due to older people being more likely to be retired and less likely to be working full or part time and unemployed. Differences across the groups in education, ethnicity, spiritual beliefs, and home duties were independent of differences in age. Thus, all (except for occupational status) were used as covariates along with age in the ANCOVA analyses. After controlling for the demographic factors, the results revealed no significant differences in mean agreement across the two samples in all but one of the 15 scenarios (see Table 1 for the adjusted means across samples). The exception was for human embryos for research purposes, where support was significantly, though only slightly higher (*p* = 0.028) for the CATI (*M* = 2.63, *SE* = 0.04) than OLP sample (*M* = 2.48, *SE* = 0.05). Given the lack of differences it was decided to combine the two samples for all subsequent analyses.

**Table 1:** Adjusted mean support and percent unsure for all scenarios across CATI and OLP samples.

	**CATI**				**OLP**				
**Application**	**Cell type**	**Mean**	**SE**	***n***	**Unsure %**	**Mean**	**SE**	***n***	**Unsure %**
Improve Health or prevent disease	Embryo	3.07	0.04	450	7.2	2.97	0.04	451	10.3^*^
	Germ	3.15	0.04	465	4.0	3.06	0.04	465	7.6^**^
	Somatic	3.21	0.03	475	1.8	3.15	0.03	479	4.8^**^
Research purposes only (Human Cells)	Embryo	2.63	0.04	453	6.4	2.48°	0.05	428	14.9^***^
	Germ	2.82	0.04	444	8.6	2.80	0.04	445	11.5
	Somatic	2.85	0.04	459	5.2	2.90	0.04	462	8.2
Research purposes only (Animal Cells)	Embryo	2.80	0.04	454	6.2	2.75	0.04	436	13.3^**^
	Germ	2.85	0.04	466	4.0	2.81	0.04	450	10.5^**^
	Somatic	2.83	0.04	459	5.2	2.85	0.04	457	9.1^*^
Change Appearance (Human cells)	Embryo	1.74	0.04	474	2.6	1.84	0.04	468	7.0^*^
	Germ	2.01	0.04	446	8.0	2.12	0.04	449	10.7
	Somatic	1.97	0.04	466	4.2	1.90	0.04	465	7.4
Animals for human purposes (food)	Embryo	2.41	0.04	435	10.2	2.38	0.04	440	12.5
	Germ	2.41	0.04	442	8.6	2.40	0.04	442	12.1^*^
	Somatic	2.28	0.04	451	6.8	2.29	0.04	446	11.3^**^

*Means are adjusted for age, education, ethnicity, spiritual beliefs and home duties. ° = ANCOVA results revealed mean was significant from CATI sample at p < 0.05; SE, Standard Error; Range for attitude, Strongly Disagree = 1, Disagree = 2, Agree = 3, Strongly Agree = 4*.

* *Results of binary logistic regression predicting unsure compared to provided an answer across OLP and CATI samples (controlling for age, education, ethnicity, spiritual beliefs and home duties) significant at p < 0.05*,

** *p < 0.01*,

*** *p < 0.001*.

Table 1 suggests that, for both samples, support dropped markedly for applications involving altering human cells to change appearance and altering animals for human purposes. All means for both samples were below the midpoint of 2.5 for these applications, and above for all other applications. Support appears particularly high for applications aimed at improving human health across all three cell types. The OLP was significantly more likely to select unsure to nearly all scenarios apart from those involving editing of germ and somatic cells for human research purposes and changing appearance, and editing embryos of animals for human purposes. This was after controlling for the effects of all covariates in a binary logistic regression, comparing the probability of being unsure relative to providing an answer across the OLP and CATI samples. This is likely due to a modality difference between the online and phone surveys where “unsure” was presented as a response option for the OLP but not for CATI (although it was accepted if a respondent stated to an interviewer that they were unsure).

### Predicting Attitudes

To assess change in attitude across cell type within each application, a multilevel model approach (Bryk and Raudenbush, 1992; Goldstein, 1995; Longford, 1995 using MLWIN Version 1.10; Rasbash et al., 2001) was employed. Two analyses were undertaken, the first to assess whether support for gene editing differed across cell type and application separately (i.e., main effects), and whether variation across cell type depends upon the aim (i.e., interaction effects). The second set of analyses examined the ability of the demographic and individual differences factors to predict variation across cell type. Both analyses utilized a three level model where variation in the outcome variable (gene editing support) across respondents was level 3, variation across applications was level 2, and variation across cell type was level 1. Unsure responses in all models were treated as missing.

### Moral and Hereditary Concern Across Gene Editing Applications

Table 2 contains the results of the 3-level model that examined the main effects of cell type and application as well as their interaction on attitude. The reference category for cell type was germ cell, meaning the parameter estimates for the predictor embryo compared support for embryos to germ cells (indicating moral concern), and those for somatic cell compared support for germ cells with somatic cells (indicating hereditary concern). Application predictors were constructed to reflect deviation contrasts or change in support across a specific aim (e.g., Improving health) compared to all other aims. Cell type by application interactions were used to assess whether any change in support across cell type depended on the gene editing application. Separate models were computed for each application (using deviation contrasts) and the results are shown in Table 2.

**Table 2:** Parameter estimates for predicting support for gene editing across application and cell type.

**Effect**	**Null model**	**Model 1 (cell type)**	**Model 2 (health)**	**Model 3 (human research)**	**Model 4 (animal research)**	**Model 5 (appear-ance)**	**Model 6 (animals for food)**
Intercept	2.58(0.02)	2.63(0.02)	2.52(0.02)	2.59(0.02)	2.59(0.02)	2.78(0.02)	2.69(0.02)
**FIXED EFFECTS**							
Embryo		−0.13(0.01)	−0.14(0.01)	−0.11(0.01)	−0.15(0.01)	−0.09(0.01)	−0.16(0.01)
Somatic		–**0.02(0.01)**	−0.04(0.01)	−0.03(0.01)^*^	–**0.02(0.01)**	**0.01(0.01)**	**0.01(0.01)**
Health			0.57(0.03)				
Health × Embryo			0.06(0.02)^*^				
Health × Somatic			0.12(0.03)				
Human Res.				0.20(0.03)			
Human Res. × Embryo				−0.13(0.03)			
Human Res. × Somatic				0.09(0.03)			
Animal Res.					0.23(0.03)		
Animal Res. × Embryo					0.10(0.03)		
Animal Res. × Somatic					**0.03(0.03)**		
Enhancement						−0.71(0.03)	
Enhancement × Embryo						−0.17(0.03)	
Enhancement × Somatic						−0.13(0.03)	
Animals for food							−0.29(0.03)
Animals for Food × Embryo							0.16(0.03)
Animals for Food × Somatic							−0.11(0.03)
**RANDOM EFFECTS**							
Level 3 Respondent	0.21(0.01)	0.21(0.01)	0.22(0.01)	0.21(0.01)	0.21(0.01)	0.23(0.01)	0.21(0.01)
Level 2 Application	0.36(0.01)	0.36(0.01)	0.28(0.01)	0.35(0.01)	0.34(0.01)	0.22(0.01)	0.34(0.01)
Level 1 Source	0.26(0.00)	0.26(0.00)	0.26(0.00)	0.26(0.00)	0.26(0.00)	0.26(0.00)	0.25(0.00)
−2*loglikelihood	29570.5	29397.13	28600.65	29268.9	29255.88	27969.17	29167.65

*The fixed effects for Embryo, Somatic, Health, Food, Human Research, Animal research, appearance, and Animals for food represent main effects. Effects with “ × ” represent interactions. Standard errors are contained within parentheses. All parameters were significant at p < 0.001 unless otherwise stated*.

* p < 0.01. Bolded parameters were not significant at p < 0.05. The −2^*^loglikelihood value represents overall model fit, with lower values representing better fit. The Null model is a model that contains no predictor variables. N was 923 for all models.

**Figure 1:**
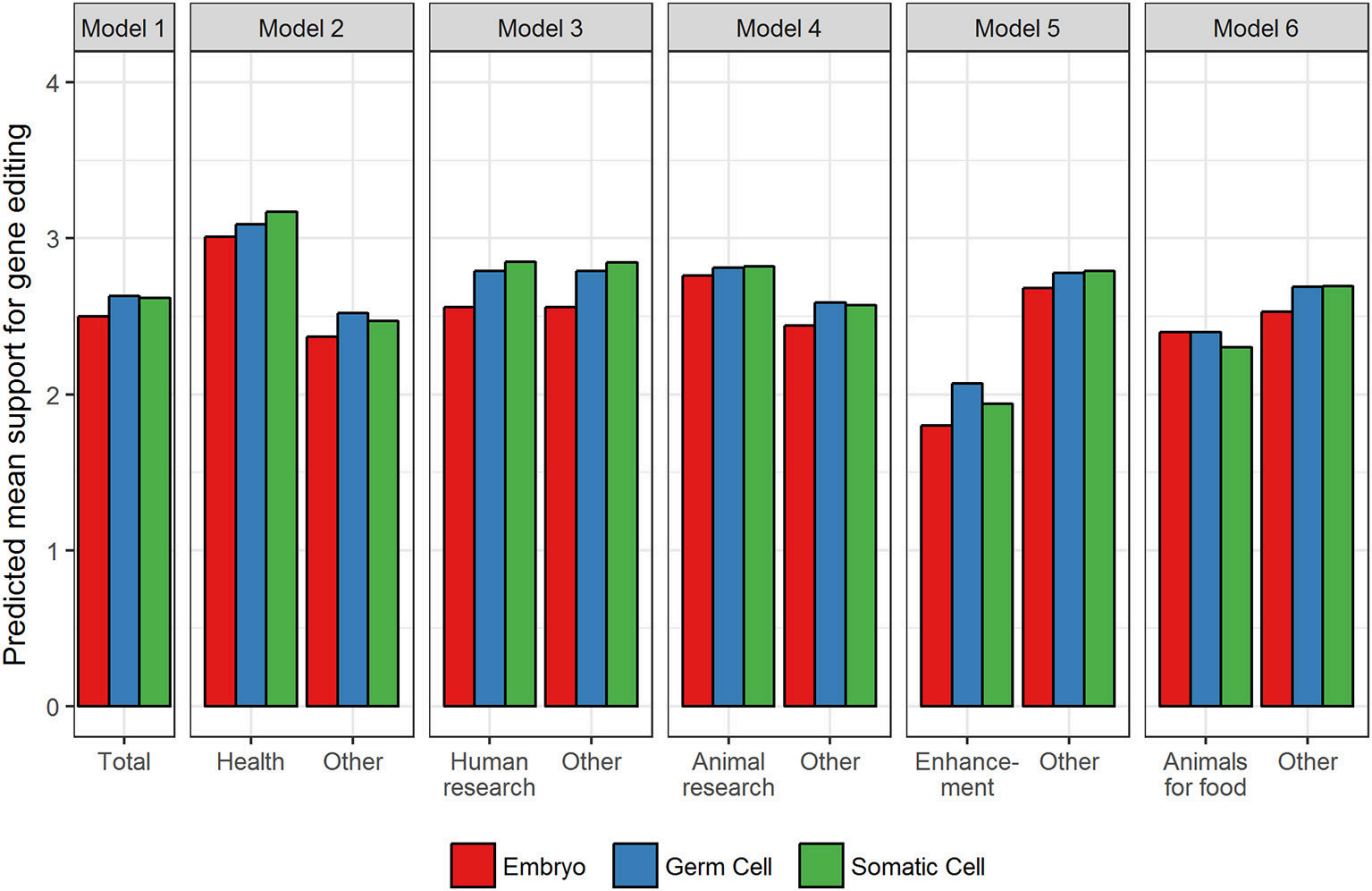
Predicted mean support for gene editing across cell type and aim. Other refers to the average of all applications minus the comparison application.

The random effects in Table 2 for the Null model reveal that there was significant variation in support for gene editing across applications and cell type and, as would be expected, across respondents. The relative size of the effects reveals that overall what gene editing is used for (i.e., the application) has a greater influence on support compared to the type of cell (0.36 vs. 0.26). In relation to the main effects for application, models 2–6 reveal that in comparison to all other applications (and averaged over cell type), support was significantly higher for improving human health, and for human and animal research (see Figure 1 for predicted means resulting from the estimates in Table 2). Support was significantly lower for enhancement and the use of animals for food. The fixed, main effect for cell type in Model 1 reveal that, averaged over all applications, there was significantly higher support for gene editing when scientists use germ cells compared to embryos, suggesting that the level of support for gene editing is dependent on moral concerns for the embryo. Model 1 also suggests that there was no significant difference between germ and somatic cells, thereby providing no evidence that hereditary concerns influence support for gene editing in general.

The pattern of results for the interaction effects, however, reveal that in some contexts, hereditary concerns may matter. When scientists use gene editing to improve human health or prevent disease, the somatic by health interaction (in Model 2) was significant. This suggests that support increases when somatic cells are used relative to germ cells when gene editing is used to improve human health, but when it is used for other purposes, the tendency for support to be higher for somatic relative to germ cells is not apparent (see Figure 1). In other words, hereditary concerns are important when gene editing is used to improve human health and for human research but not for animal research, enhancement, or using animals for food. There were no significant somatic vs. germ cell differences for animal research, but unexpectedly for human enhancement and using animals for food, the significant somatic cell interactions revealed that support was higher for germ than somatic cells (see Figure 1).

The interactions relating to embryos (relative to germ cells) were also significant across all applications. Gene editing in embryos generated significantly lower support than germ cells across all applications except animals for food, but the extent of this effect varied across applications (see Figure 1). The effect was largest for human research and human enhancement (represented by the negative interaction effect), and smallest for human health and animal research. There was no difference in support for embryos relative to germ cells for the use of gene editing in animals for food.

### Individual Differences in Moral and Hereditary Concern

The second set of analyses examined individual difference predictors of variation in support for gene editing across cell type. The first model contained the following fixed effect predictors entered simultaneously: CATI vs. OLP sample; Embryo vs. Germ Cell; Somatic vs. Germ Cell; Age, Gender, Education, Religion, Ethnicity, Gene Editing Knowledge, Political Orientation and Trust in Scientists (see Table 3 for parameter estimates). A second model added interactions between the individual difference factors and the two cell type predictors, embryo and somatic, to investigate whether moral or hereditary concern varied across different respondents. Given that hereditary concerns were only found to occur in gene editing to improve human health and human research, a third model was tested using only these two applications in the analysis. Interactions were entered one at a time in the order presented in Table 3. If the interaction was significant at *p* < 0.05 it was retained in the model. Table 3 shows the results for the final models.

**Table 3:** Parameter estimates for predicting variation in support across cell type from individual differences.

**Effect**	**Model 1**	**Model 2**	**Model 3 (human health and human research only)**
Intercept	2.20(0.09)	2.24(0.10)	2.43(0.13)
**FIXED EFFECTS**			
**MAIN EFFECTS**			
OLP	−0.01(0.03)	−0.01(0.03)	−0.03(0.04)
Embryo	−0.12(0.01)^***^	−0.12(0.05)^*^	−0.12(0.08)
Somatic	−0.02(0.01)	−0.139(0.05)^**^	−0.06(0.08)
Age	−0.002(0.001)^*^	−0.002(0.001)^*^	−0.002(0.001)^*^
Female	−0.18(0.03)^***^	−0.15(0.03)^***^	−0.07(0.04)
University educated	0.03(0.03)	0.03(0.03)	0.02(0.04)
Religion^ψ^	−0.06(0.01)^***^	−0.06(0.01)^***^	−0.08(0.02)^***^
Australian	−0.02(0.04)	−0.03(0.04)	0.05(0.05)
Knowledge	0.02(0.006)^***^	0.02(0.006)^***^	0.015(0.008)
Political orientation	0.01(0.01)	0.01(0.01)	0.01(0.01)
Trust in Scientists	0.18(0.02)^***^	0.17(0.02)^***^	0.19(0.02)^***^
**INTERACTIONS**			
Embryo × Female		−0.06(0.02)^**^	−0.09(0.04)^*^
Somatic × Female		−0.03(0.02)	−0.01(0.04)
Embryo × Religion		−0.003(0.01)	−0.037(0.015)^*^
Somatic × Religion		0.02(0.01)^*^	0.02(0.015)
Embryo × Australian		0.06(0.03)^*^
Somatic × Australian		0.08(0.03)^**^
Embryo × Trust		0.004(0.01)
Somatic × Trust		0.02(0.01)^*^
**RANDOM EFFECTS**			
Person	0.20(0.01)	0.20(0.01)	0.20(0.02)
Type	0.31(0.01)	0.31(0.01)	0.15(0.01)
Cell	0.25(0.00)	0.25(0.00)	0.26(0.007)
−2loglikelihood	25727.0	25696.6	9860.59

*Standard errors are contained within parentheses*.

ψ *Religion consisted of the mean score of church attendance, spirituality and trust in the churches (α = 0.73). The Null model was identical to that presented in Table 2, so was not reported here*.

* *p < 0.05*,

** *p < 0.01*,

*** *p < 0.001. n was 821 for all models*.

The fixed main effects for the individual difference variables in Table 3 for Model 1 suggest that attitudes toward gene editing significantly vary according to age, gender, religion, knowledge and trust in scientists. Averaged over cell type and application, higher support was evidenced amongst younger people, males, those with lower religiosity, and those with more self-reported knowledge and trust in scientists. Education, ethnicity and political orientation were not associated with general support for gene editing. The interaction effects for Model 2 averaged over all applications in Table 3 suggest that only gender and ethnicity were significantly associated with moral concern. Specifically, the predicted means (derived from the regression equation) show that males (Y′ = 2.38) and females (Y′ = 2.23) demonstrated similar support for gene editing using germ cells, but support was lower amongst females (Y′ = 2.11) than males (Y′ = 2.32) for the use of embryos. This suggests that the moral status of the embryo was more of a concern for females (${{\text{Y}}^{\prime }}_{\text{embryo}}$-${{\text{Y}}^{\prime }}_{\text{germ}}$ = −0.12) than males (${{\text{Y}}^{\prime }}_{\text{embryo}}$-${{\text{Y}}^{\prime }}_{\text{germ}}$ = −0.06). The predicted means for ethnicity also show that moral concern was stronger for Australians (${{\text{Y}}^{\prime }}_{\text{embryo}}$ [2.05]-${{\text{Y}}^{\prime }}_{\text{germ}}$ [2.26] = −0.21) compared to non-Australians (${{\text{Y}}^{\prime }}_{\text{embryo}}$ [2.11]–${{\text{Y}}^{\prime }}_{\text{germ}}$ [2.31] = −0.12).

**Figure 2:**
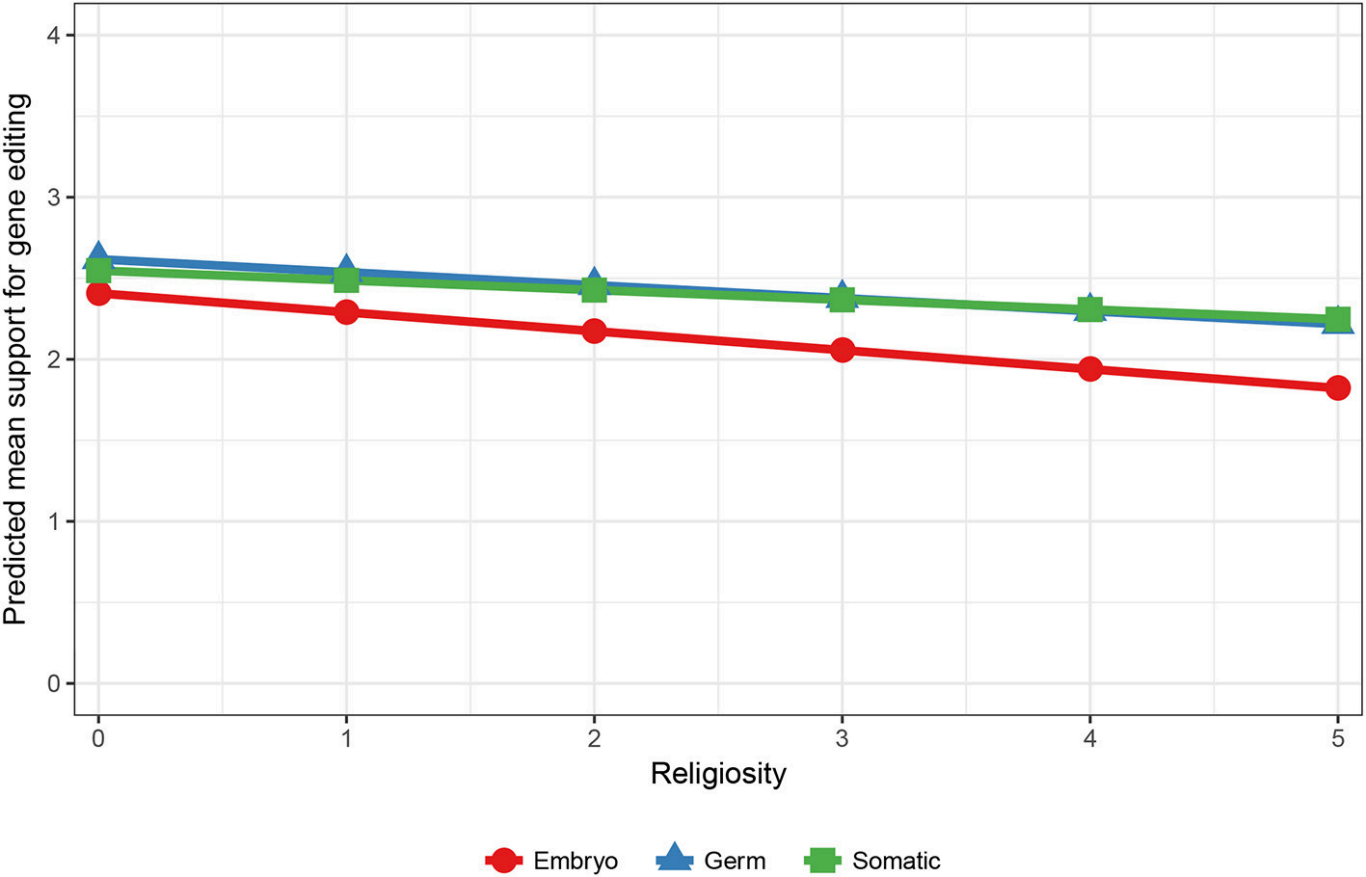
Predicted mean support for gene editing across cell type and religiosity. 5 = Higher religiosity.

Interestingly, when only the applications of improving human health and human research were analyzed, the interaction effects in Model 3 show that moral concern still differed across males and females, but also across different levels of religiosity. An effect for ethnicity was no longer significant, suggesting that Australians demonstrate greater moral concern overall, but not when the purpose of the editing is to improve human health or human research. The predicted means for gender show that moral concern was stronger for women (Y′_embryo_ [2.29]–Y′_germ_ [2.54] = −0.25) compared to men (Y′_embryo_ [2.45]–${{\text{Y}}^{\prime }}_{\text{germ}}$ [2.61] = −0.16) when the purpose of gene editing is for human health and research. The predicted means for religion showed that as religiosity increased the gap between support for using embryos compared to germ cells increased (see Figure 2). Thus, greater moral concern was associated with stronger religiosity when gene editing is used to improve human health and human research.

In relation to hereditary concern, the interaction effects in Model 2 in Table 3 reveal that variation in support across somatic vs. germs cells was significantly associated with ethnicity, trust, and religion. Interestingly, this was only the case when all gene editing applications were included in the analysis. When only human health and research were examined, there was no evidence for hereditary concerns either across the entire sample or amongst specific individual difference groups. The predicted means for ethnicity (using the coefficients from Model 2) showed a reversed hereditary concern effect, in that averaged over all aims, support for germ cells was higher than somatic cells. This was the case for both Australians and non-Australians, but the difference was greater for Australians (0.13) than non-Australians (0.05). The predicted means for the effect of religiosity revealed that hereditary concern increased alongside stronger religiosity. For example, for those who scored zero on religiosity, support for germ cells was greater than somatic cells (0.08). This difference however decreased with stronger religiosity, disappearing for those who scored on the extreme end of the scale (i.e., religiosity = 5) (−0.03). The effect for trust in scientists showed higher predicted support for germ relative to somatic cells for those demonstrating low levels of trust (i.e., trust = 0) (0.07), whilst slightly higher support for somatic relative to germ cells was estimated for those reporting high levels of trust (i.e., trust = 5) (−0.04).

## Discussion

In general, the results of this Australian survey correspond with US and European findings, suggesting public support for gene editing depends on the application (McCaughey et al., 2016; Gaskell et al., 2017). That is, support is relatively high when the purpose is to enhance human health, and low when the purpose is to enhance attributes or appearance in otherwise healthy individuals. Extending on existing knowledge, we also found the sample supported editing genes within the context of animal as well as human research, and that both generated significantly more support than enhancing human appearance and animal attributes for the purpose of improving human food. These results coincide with general research suggesting that Australians demonstrate consistently high levels of trust in scientific research (Bruce and Critchley, 2003–2017), general support for the use of animal models in medical research (under certain conditions) (Critchley et al., 2013) and a distaste of designer babies and genetically modified animals for food (Marques et al., 2015). They also validate the findings generated from recent online panel studies, given the similarities we found between our OLP and CATI samples (after statistically controlling for demographic differences).

### Moral Concern Associated With Editing Embryos

In line with other recent studies (Critchley et al., 2013; Treleaven and Tuch, 2018) our results suggest that Australians are relatively comfortable with the idea of editing human and animal embryos, but only for research purposes or to enhance human health. Editing human and animal embryos for the purpose of enhancing human or animal attributes, respectively, was not supported. Thus, this study aligns with earlier research suggesting that Australians are generally supportive of embryonic research if it will lead to cures for serious human disease (Critchley and Turney, 2004; Critchley, 2008). Moral concern was however found to occur in all applications assessed by this research except for editing animal embryos for the purpose of improving human food. The low support for this application combined with the absence of a moral concern effect suggests the influence of other factors.

Strong public opposition to genetically modified animals for food has been consistently demonstrated within Australia (Bruce and Critchley, 2003–2017; Marques et al., 2015) and elsewhere (Schuppli et al., 2015), and has often been attributed to unease associated with safety, animal welfare and low trust in regulators (Kronberger et al., 2014; Shriver and McConnachie, 2018). It is possible, therefore, that these factors may override the influence of moral concern for animal embryos when the purpose is for human food. Moral concern did however reduce support in the context of using animals for research. Perhaps moral concern in a research context reflects concern with animal welfare rather than safety or lower trust which are more likely to occur within the context of genetically modified animals for food.

In the two research contexts, the impact of moral concern on support for gene editing was stronger for human compared to animal embryos. While this may reflect a value orientation emphasizing the importance of human life over all other forms (at least for those opposed to embryonic research more generally) (Kass, 1997), it does not support the findings of a national survey of 2700 Australians that stem cell research using animal embryos generated statistically equal levels of comfort (or discomfort) as human embryos (Critchley et al., 2013). The fact that moral concern was also less important for the application of enhancing human health than the human research context may provide a clue to this inconsistency. The human research condition was articulated to respondents as simply, “do you agree with editing the genes of human embryos for research purposes only,” while the enhancing human health condition was, “human embryos to improve health or prevent disease.” The question relating to human research could have been interpreted as including multiple purposes (including some that are potentially undesirable such as cloning) resulting in higher concern for the embryo than when health benefits are directly promised, and where ethical concerns are more likely to be compromised (Caulfield et al., 2006).

Similarly, an underlying willingness to compromise ethical or moral principles if research is likely to cure disease may have also explained the lower moral concern for animal embryos in the research context. In the Australian study where similar support was found for research using animal (i.e., mice) and human embryos (Critchley et al., 2013), respondents were clearly informed in both contexts that the aim of the stem cell research was to find cures for untreatable diseases in humans. The only other animal scenario included in this research was the unpopular application of editing animal cells for the purposes of human food. With the negative food application statistically controlled for, the research purposes for animals could have been perceived to include research that was beneficial for animals as well as humans. Because no question assessing specific health benefits using animals was included, this was not controlled for as it was for human research. Thus, it was possible that animal research was perceived to include more beneficial purposes on average than human research (as preventing disease via human editing was accounted for). Clearly future research is needed to confirm this finding by also including a scenario where the use of gene editing within animals is described as being conducted specifically to improve human health or animal health and welfare.

### Hereditary Concern

Previous studies have confounded hereditary and moral concerns by only using embryos (or unborn children) to compare with somatic or adult cells (McCaughey et al., 2016; Gaskell et al., 2017). The design of this study allowed for the separating of these two effects to confirm for the first time, that public concern about the moral status of the embryo is independent from any hereditary concern. Although moral concern was found to be more pervasive than hereditary concern, the latter was found to be present for applications involving humans. Averaged over all applications there was no significant difference in support between cells that were specifically described as being able to transmit edits to offspring (i.e., germ cells) and those that could not (i.e., somatic cells). However, when human genes are edited for the purpose of improving human health or for human research, support for the use of germ cells was significantly less than somatic cells. Gene editing animals for research or human purposes did not generate concern for edits being passed on to future generations. Although future research is needed to examine the reasons for these findings, a possible explanation for the lack of hereditary concern amongst animal applications is that the breeding and behavior of domesticated research and farm animals is tightly regulated within Australia, arguably posing little threat that their modified genes will be transferred to the wider ecosystem.

This is inconsistent however, with earlier research concluding people are not comfortable with the possibility of genetically engineered animals passing on harmful defects to offspring (Schultz-Bergin, 2018). This would suggest hereditary concern due to fear for the offspring's welfare rather than detrimental safety or environmental effects. If moral concern was associated with welfare concern, then any worry about edited animals passing on harmful effects to offspring may have been removed when the effects of moral concern on gene editing support were accounted for (at least in the animal research application). Future research should therefore assess support for gene editing within animals in additional contexts that directly specify the potential for harmful consequences to the animal's offspring (e.g., the type of gene edited and resulting phenotype) as well as the environment (e.g., gene editing in animals that are not in captivity). Comparing reactions to the editing of non-domesticated animals would also be helpful, particularly those whose breeding could permeate the wider ecosystem.

### Individual Differences in Support for Gene Editing

Averaged over cell type and application, the current research found significantly higher support amongst males, younger people, and those reporting lower religiosity, more knowledge and stronger trust in scientists. This supports the work of McCaughey et al. (2016) as well as Weisberg who also found amongst a sample of social media users and online panel members, respectively, higher support for gene editing amongst males and younger respondents. Gaskell et al. (2017) also found higher support amongst males, but age was not associated with attitudes. In line with the findings of McCaughey et al., we also found that those with lower religiosity were more supportive. In contrast to previous studies however (McCaughey et al., 2016; Weisberg et al., 2017), and similar to the results reported by Gaskell et al., higher support was not found to be associated with higher education. Finally in contrast to Weisberg, a more left wing political ideology was not associated with more support in this study.

Possible explanations for the demographic differences in attitudes to gene editing in general are likely to be complex involving psychological, socio-political and cultural factors (Hartman et al., 2017). Indeed there is still debate within the general attitudes toward science literature about why certain demographic differences influence attitudes. The complexity is also compounded by the different measures, analyses techniques and combination of demographic variables used across studies (Sturgis and Allum, 2001). For example Weisberg et al. found that support for gene editing rose for those with a more left wing political ideology but did not measure or control for religiosity. We controlled for both of these correlated variables (Rutjens et al., 2017) and found religiosity rather than political orientation was important. Thus, understanding why certain demographic characteristics may or may not influence attitudes to gene editing also requires much more research including direct comparisons of cultural or country differences in the importance of predictors (especially religion and political orientation), as well as comparisons across different types of samples. Indeed our research demonstrated extensive differences between the demographic background of the OLP and CATI surveys, which could further alter the impact of certain demographic variables on support. Despite this, our results add to previous findings that males, younger respondents and those with more education are more supportive of gene editing. Given that higher support for science and emerging biotechnologies (including GM foods) in general have also often been found amongst these groups (Rutjens et al., 2017), future research should also investigate whether gene editing is viewed differently from other biotechnology applications amongst different groups or whether this is simply a reflection of support for science in general (Hartman et al., 2017).

In relation to moral concern for embryos, it was found that women and those who were Australian born displayed significantly more unease when all applications were considered. However, when the application involved only human embryos (for human health enhancement and human research) the effect remained for gender but disappeared for ethnicity, and became significant for those with stronger religiosity. This suggests that women are more concerned than men about embryonic editing regardless of its purpose, and that Australian born respondents are only more concerned in applications involving animals and enhancing human attributes. In support of previous research indicating religious beliefs are associated with lower support for embryonic research (Nisbet, 2005; Ho et al., 2008; Critchley and Nicol, 2009; McCaughey et al., 2016) increasing discomfort with editing embryos was displayed as religiosity increased. The fact that religion did not significantly predict increased moral concern for embryos when the application involved animals or enhancing human attributes provides support for the idea that even when the editing is being conducted to cure or prevent disease, human embryos are considered sacred and morally distinct from animal embryos for religious individuals.

The findings associated with gender and moral concern coincide with previous findings that women are generally less accepting of biotechnology and science in general than men. The reasons for more general gender differences are not clear and have been attributed to women's lower interest, knowledge and educational opportunities (Hayes and Tariq, 2000), decreased trust in science and increased religiosity compared to men. Some scholars have also suggested that gender differences are due to men attributing more importance to science due to stereotypical beliefs associating science with male rather than female attributes (Rutjens et al., 2017). While we cannot assess differences due to stereotypical beliefs, gender effects in this study were still found to significantly reduce support for editing embryos (in all applications) after removing the effects of knowledge, trust, education and religion, thereby suggesting alternative explanations.

Interestingly, Simon (2012) argues that women are less supportive of biotechnology because they are more likely than men to be directly affected by any negative consequences. Potentially harmful outcomes of new and untested biotechnological developments have more direct implications for women's bodies, their reproductive processes, offspring and the selection and preparation of food. Feminist scholars also point out that women's bodies are often at the center of biotechnological controversies, especially reproductive technologies which are at particular risk of increasing commodification of the human body (Kirejczyk, 2008). This may further generate an emotive “yuck factor” type response amongst women providing another source of discontent to explain their decreased support for research involving embryos. These explanations remain speculative however, as the complex reasons for gender differences in relation to concerns for human and animal embryos need to be further investigated. There are also other possible reasons for gender differences, such as women being more sensitive to genetic inequality given their historical oppression by men. Research needs to directly capture the underlying values, emotions, motivations and world views of both men and women, and how they may interact with other contextual factors to drive gender differences in support for gene editing.

There were no gender differences in hereditary concern which is interesting given women's consistent reduced general support relative to men for a range of biotechnologies. Perhaps the description of germ cells could have been attributed to either gender effectively removing Simon's (2012) gender salience effect. General hereditary concern was however significantly associated with ethnicity, trust and religion. Possibly reflecting a general distaste toward scientists playing god, higher religiosity was associated with greater hereditary concern. The result for ethnicity was intriguing as both Australians and non-Australians demonstrated a reverse hereditary effect in that editing germ cells were viewed as more positive than somatic cells, but the effect was even more pronounced for those reporting an Australian identity.

After controlling for all demographic and individual differences as well as moral concern and any associated welfare concerns, the reverse hereditary effect may indicate that editing germ cells is perceived to be more beneficial than containing an edit within a somatic cell. Improving human attributes, removing diseases or improving animals for food, if safe and not morally objectionable (and conducted by trusted scientists), would have more impact if unwanted characteristics are no longer passed onto future generations. The phrasing of the information provided to respondents before they answered any questions emphasized the ability of gene editing to prevent the inheritance of disease and increase resistance. This may have primed some respondents to view the passing on of desirable edits as positive rather than negative, supporting Shriver and McConnachie's (2018) notion that new forms of gene editing (especially within animals) may be viewed as more acceptable due to not mixing species and a reduction in off-target edits resulting in harmful consequences. They further argue that increasing an understanding amongst the public of these issues may therefore increase support for editing germlines in some contexts if it were used in the interests of animal and human welfare as well as the environment.

Clearly more research is needed to unravel why hereditary concern may or may not translate into support for germline editing. After statistically removing the effects of moral concern, trust and religion, Australians were curiously even more supportive of germline editing than other ethnic identities. An explanation for this interesting result may be that Australian ethnic identity could have acted as a proxy for other factors associated with a preference for germ cells, such as increased exposure to positive media portrayal of the benefits of gene editing. But this is purely speculative and difficult to determine given the vast array of ethnic identities contained in the non-Australian category and the absence of measuring media exposure in this study. It is also curious that increased knowledge was associated with support overall, but was not associated with reversed hereditary concern. If increased knowledge included an awareness of the ability of more recent gene editing tools to reduce ethical and safety concerns it should be associated with more support for editing germ cells. Clearly what self-reported knowledge (as measured in the current study) is measuring needs to be examined, and more objective measures of knowledge across a number of areas of gene editing should be constructed, as well as instruments to capture the extent and type of media exposure.

Another explanation for the reversed hereditary finding is that it may be an artifact of a methodological effect. Support for gene editing measures were presented to respondents in two sections. The first described edits that can be passed onto offspring and contained the embryo and germ cell questions together. The second section contained only the scenarios with somatic cells. By placing the embryo and germ cells together a contrast bias may have occurred resulting in inflated support scores for germ cells. The more emotionally charged embryo examples may have been used as the perceptual anchor in which to evaluate or compare the germ cell applications. If an individual views embryo editing as undesirable, they may perceive the less negative comparative option (i.e., germ cells) to be more positive than they normally would (Skowronski and Carlston, 1989). In other words to emphasize their negative orientation toward embryos higher support is attributed to the apparent opposition which was germ cells. Because negative behaviors or attributes are more likely to be used as the perceptual anchor (Skowronski and Carlston, 1989), and perceptions of them are less resistant to change (Baumeister et al., 2001), it is more likely that an increased positivity bias would be in effect for germ cells (Wänke, 1996) rather than an inflation of disapproval for embryos. This could therefore explain why support was higher for germ rather than somatic cells in some applications.

Even higher support for germ relative to somatic cells was found for those lower in trust. This is counter intuitive given that it would be expected that those who do not trust scientists would be more concerned about edits being passed on (if harmful). In line with previous research in the wider biotechnology literature, higher trust was strongly associated with greater support for gene editing across all applications and cell types. After controlling for all demographic and individual difference variables and the significant interactions between the somatic/germ comparison and ethnicity, religion and gender, the effect of trust on hereditary concern may have inadvertently captured a susceptibility to a contrast bias. After accounting for religion and gender which were also associated with hereditary concern, lower trust in scientists may have represented a range of factors responsible for an increase in bias such as a general tendency for skeptical individuals to polarize comparisons (Gunther, 1988), decreased cognitive engagement with the survey questions (Bukovskaya and Shmukler, 2016) or susceptibility to cognitive biases in general (Spithoven et al., 2017). It would therefore be beneficial for future research to reduce the potential for contrast effects by using a between groups design where different individuals are presented with each cell type and application.

## Conclusions

This study has revealed that the nature of support for gene editing is complex and determined by the application, the type of cell and also individual differences. Importantly, it has revealed for the first time, that concerns about edits being passed onto human offspring are present alongside those associated with the moral status of the embryo. It also suggests, albeit tentatively that germline editing may not be controversial amongst some members of the public if moral (and possibly other) concerns are dealt with. One of the most important ethical issues arising from the gene editing community is the risk of passing on unintended or detrimental genetic modifications to future generations. This is distinct from the use of embryos in research more broadly, and as such requires a different approach to public education and engagement. It also arguably supports the implied recommendations of the International Summit on Human Gene Editing that called for distinct regulatory responses to ensure the protection of future living organisms and the environment, as well as the protection of the embryo at one point in time. Current bans in some countries on all germline editing may be considered by a large majority of the public to be too restrictive. Our result that hereditary and moral concern are independent therefore suggests that policy makers and regulators keen to accommodate public opinion should consider carefully the separate issues involved with editing embryonic compared to other types of germ cells.

The editing of somatic cells was found to be supported in all applications assessed by this research apart from editing the genes of animals for human use. This suggests that the likelihood of public opposition to editing is low within the clinic where therapeutic developments by altering the genetic information responsible for Mendelian diseases is currently most promising. Though lower in some applications, support for editing cells within gametes was also generally supported, though again not within animals for human purposes. Although more research is needed to validate the findings, this study implies that the public do support edits that can be passed on to future offspring and that this support may increase alongside an awareness of new methods such as CRISPR-Cas9. That is, specific knowledge relating to CRISPR-Cas9's potential to reduce harmful off-target edits and not needing to introduce the genetic material from one species into another. Education and engagement strategies targeting religious individuals and non-Australians may also increase this support further.

## Data Availability Statement

Datasets are available on request—the raw data supporting the conclusions of this manuscript will be made available by the authors, without undue reservation, to any qualified researcher.

## Ethics Statement

This study was carried out in accordance with the recommendations of the National Statement on Ethical Conduct in Human Research, National Health and Medical Research Council (Australia) with informed consent from all subjects. The protocol was approved by the Swinburne University Human Research Ethics Committee.

## Author Contributions

CC and DN conceived the study. CC, DN, GB, and JW contributed to the design of the survey instruments. GB and JW collected the data. CC drafted the manuscript and analyzed the data. All authors made a substantial contribution toward the development of the final manuscript and approved publication.

### Conflict of Interest Statement

The authors declare that the research was conducted in the absence of any commercial or financial relationships that could be construed as a potential conflict of interest.

## Footnotes

^1^Although this study also assessed attitudes towards genetically modified food, the level of agreement for the question, “How much do you agree with the use of genetically modified food?” was not reported.

^2^Prior knowledge of gene editing was also shown to be significantly associated with support for the two adult and children disease conditions, but the direction of the effect could not be ascertained as the question or coding of responses for this question were not available.

^3^The risks stated were: For individuals, it could have unintended consequences, or lead to unexpected mutations. For society, it could lead to eugenics.

^4^Qualtrics online panels. Available online at: https://www.qualtrics.com/au/online-sample/

^5^Sampleworx.
