# Supplementary material for: Predicting Public Attitudes Toward Gene Editing of Germlines: The Impact of Moral and Hereditary Concern in Human and Animal Applications
Source: Front Genet. 2019 Jan 9;9:704. doi: 10.3389/fgene.2018.00704 (PMC6334182; doi:10.3389/fgene.2018.00704)
Supplement: Supplementary file 1 [file Data_Sheet_1.PDF]

Swinburne National Technology and Society Monitor 2017

Hello, my name is [name] and I'm calling from Swinburne University in Melbourne. We're not selling anything - we're conducting a survey for the university concerning people's attitudes towards new technologies and science. We've done the survey every year since 2003, and this year we are interested in what Australians think about editing genes. It takes about 10-15 minutes. Would you like to be involved?

Are you 18 years of age or older?
1. Yes (Go to Privacy Statement)
0. No (Go to next question)

IF NO:
Is there someone else at home who is 18 years of age or older?
1. Yes (Go to next question)
0. No (Failed screen – Go to end)

IF YES:
May I speak with him or her?"
1. Yes (Go back to Introduction for person over 18)
0. No (Failed screen – Go to end)

PRIVACY STATEMENT

Before we begin, I want to let you know that the information you provide is strictly confidential. We have no record of your name and address and you can stop the interview at any time if you do not wish to continue. Results from this survey will only be used for research purposes and may be published in scientific journals. Only group information is published so that no one can be identified. If at any time during the survey, you're not sure, then just say unsure.

SOCIAL ISSUES

What issue(s) or social problem(s) do you find to be the most important in Australia today? Please limit your answer to three issues/social problems.
[open response]

GENERAL COMFORT

I would now like to ask you how comfortable you are with different types of technologies. Using a scale from zero to 10, where zero means 'not at all comfortable', and 10 means 'very comfortable', in general how comfortable are you with the rate of technological change in the world today?"

SPECIFIC TECHNOLOGIES

More specifically, using the same scale from zero-10, how comfortable are you with:

RANDOMISE

- The internet (in general)
- Mobile phones
- Genetically modified plants for food
- Genetically modified animals for food
- Nuclear power plants in Australia
- Genetic Testing (generally)
- Cloning human babies
- Stem cell research using left-over IVF embryos
- Stem cell research using tissue from adults
- Drugs for reducing depression or anxiety
- Psychological therapy for depression and anxiety
- Vaccinations in general

GENE EDITING

In this year's survey we are interested in your views on gene editing.

On a scale of zero to 10, where zero means 'I know nothing about gene editing' and 10 means 'I know a great deal about gene editing', how would you rate your current knowledge?

I am now going to read out a description of gene editing.

Genome editing is when scientists deliberately alter the genes in a living cell to change how a gene functions.

So far, most uses of gene editing have been for research purposes only, however there are many possible applications. For example, it could be used to alter the genes of plants and animals to increase resistance to disease. It could also be used to develop new drugs, prevent the inheritance of diseases, and determine the attributes of babies.

We are interested in what you think about the use of gene editing for a range of purposes. I will read out a number of scenarios and ask you for your level of agreement with each. The response options are Strongly Disagree, Disagree, Agree and Strongly Agree. If you are unsure just say unsure.

The first lot of scenarios relate to editing genes that could potentially lead to the change being inherited (passed onto another human or animal). "

RANDOMISE

- Human embryos to improve health or prevent disease (e.g., cancer)
- Human embryos for reasons of producing a baby with certain genes (e.g., for hair colour, gender selection)
- Human embryos for research purposes only
- Human reproductive cells (egg or sperm) for research purposes only

- Human reproductive cells (egg or sperm) to improve health or prevent disease (e.g., cancer)
- Human reproductive cells (egg or sperm) for reasons of producing a baby with certain genes
- Animal embryos for research purposes only
- Animal reproductive cells (egg or sperm) for research purposes only
- Animal embryos for human purposes (e.g., improving the quality of beef)
- Animal reproductive cells (egg or sperm) for human purposes (e.g., improving the quality of beef)

The next lot of scenarios relate to editing the genes within a somatic/body cell that cannot pass on the change to another human or animal. The response options are Disagree strongly, disagree, agree and agree strongly. If you are unsure just say unsure.

For each of the following again let us know the extent to which you agree or disagree with editing the genes of:

RANDOMISE
- A human body cell (e.g., eye or heart cell) to improve health or prevent disease (e.g., blindness)
- A human body cell to change one's appearance
- A human body cell for research purposes only
- An animal body cell to alter its appearance (e.g., coat patterns in cats or dogs)
- An animal body cell to alter its characteristics for human purposes (e.g., leaner beef in cows)
- An animal body cell for research purposes only

<u>TRUST</u>
Now some questions about people and organizations that you might depend upon for information about new technologies. I'd like to know how much you trust the following people and organizations on a scale from zero to 5, where zero means 'don't trust at all', and 5 means 'trust a very great deal.

RANDOMISE
- The Federal government
- Major Australian companies
- Major international companies
- Universities
- The commercial media
- The non-commercial media (e.g., ABC)
- The State government
- The churches (in general)
- The environmental movement
- The public service
- Scientists
- CSIRO (the Commonwealth Scientific and Industrial Research Organization)
- Trade unions

- Hospitals
- Psychiatrists
- Psychologists

SCIENCE IN GENERAL

Now I would like you to tell me how much you agree or disagree with the following statements about science and technology. Using a scale from zero to 10, where zero means 'strongly disagree', and 10 means 'strongly agree'.

RANDOMISE

- Scientists have too much control over nature.
- Science and technology can solve most problems faced by human beings.
- Science and technology are continuously improving our quality of life.
- It is important for governments to regulate new technologies.
- Science and technology are out of control, and beyond the control of governments.

WELL BEING

The last lot of questions relate to how you have been feeling lately. We are interested in how peoples' stress levels can influence their opinions in relation to health sciences. I will read out a range of feelings and would appreciate if you could say how often you have experienced each in the last 4 weeks. For each question, please respond with one of the following options: None of the time, A little of the time, Some of the time, Most of the time, or All of the time.

During the past FOUR weeks , how often did you feel…?

- Tired out for no good reason
- Nervous
- So nervous that nothing could calm you down
- Hopeless
- Restless or fidgety
- So restless you could not sit still
- Depressed
- That everything was an effort
- So sad that nothing could cheer you up
- Worthless

DEMOGRAPHICS

That's the end of the questions, but before we finish I need to ask you for some demographic information.

Employment Status

What is your current employment status?
[INTERVIEWER PROMPT: If respondent unsure ask them to choose the one that most applies to their current situation]

1. Work full time
2. Work part time
3. Home duties

4. Retired
5. Unemployed
6. Other (Specify)

Occupation

What is your occupation? (open)

[If retired/unemployed/home duties]
What was your occupation before you retired/became unemployed?

Education

What is the highest level of education you have completed?

1. Less than Year 12 Secondary School
2. Year 12 Secondary School
3. TAFE diploma or certificate
4. University degree or diploma
5. Postgraduate degree

Church Attendance

Apart from special religious ceremonies such as weddings and funerals, how often do you attend religious services?"

0. Never
1. Less than once a year
2. At least once a year
3. Several times a year
4. At least once a month
5. At least once a week

Year Born

In what year were you born?

Gender

What is your gender?

1. Male
2. Female
3. Other

Political Orientation

In political matters, people talk about the left and the right . On a scale from 1 to 10, where 1 = Left and 10 = right, where would you place your views generally speaking.?

[INTERVIEWER PROMPT: If people ask for clarification, please read the following:

People who describe themselves as left wing typically want to change traditional and cultural customs rather than preserve them. They also strongly believe in principles such as government intervention rather than individual responsibility, distributing wealth and private property equally among all people, and challenging authority.

People who describe themselves as right wing typically want to preserve traditional and cultural customs rather than change them. They also strongly believe in principles such as individual responsibility rather than government intervention, the accumulation of wealth and private property, and respect for authority]

Postcode

What is your post code?

END

Thank you very much for participating in this survey. We appreciate you giving your time, and your contribution has been very valuable. Would you like any further information about the project or a contact number for any complaints?

[Research ethics contact details]

Would you like any further information about the project or a contact number for any complaints?

[CI Contact details]

That is the end of the survey. Thank you very much for participating. We appreciate you giving your time, and your contribution has been very valuable.
